# Effects of topical mitomycin-C on the tracheal epithelia of rabbits following tracheostomy

**DOI:** 10.3906/sag-1802-187

**Published:** 2021-04-30

**Authors:** Saniye Göknil ÇALIK, Mustafa ÇALIK, Zümrüt Ela ARSLAN KAŞDOĞAN, Mustafa Cihat AVUNDUK, Olgun Kadir ARIBAŞ, Hıdır ESME

**Affiliations:** 1 Department of Emergency and First Aid, Vocational School of Health Services, KTO Karatay University, Konya Turkey; 2 Department of Thoracic Surgery, Konya Training and Research Hospital, Health Sciences University, Konya Turkey; 3 Department of Anesthesiology and Reanimation, Health Sciences University, Konya Training and Research Hospital, Konya Turkey; 4 Department of Pathology, Meram Faculty of Medicine, Necmettin Erbakan University, Konya Turkey; 5 Department of Thoracic Surgery, Faculty of Medicine, Gazi University, Ankara Turkey

**Keywords:** Tracheostomy, trachealepithelia, rabbits, mitomycin-C(MMC)

## Abstract

**Background/aim:**

We aimed to investigate the topical application of mitomycin-C (MMC) after the conventional tracheostomy in a rabbit model.

**Materials and methods:**

Twenty-four male New Zealand White rabbits were randomly divided among 3 equal groups (n: 8). Tracheostomies were performed on 16 subjects. Group 1 which served as a control for all tracheal measurements. After tracheostomy, we applied sterile saline (group 2) or MMC at 0.8 mg/mL (group 3) around the tracheotomy site for 5 min. At the 3rd week after surgery, all tracheas were subjected to morphometric and histopathological examinations, including tracheal lumen diameter (LD), number of capillary vessels (CV), subepithelial tissue thickness (SETT), fibroblasts, and inflammatory cells (IC).

**Results:**

There was a statistically significant difference between the two tracheostomy groups themselves and the control group for LD (p = 0.035), CV (p = 0.006), SETT, fibroblasts, and IC (p < 0.001). Histopathological analysis showed the decreased LD, CV, SETT, IC, and fibroblasts compared to MMC with tracheostomy groups. MMC was more effective than saline for LD, CV, SETT, IC, and fibroblasts.

**Conclusion:**

Wound healing modulation may prevent scar formation. Fibrosis decreased following tracheostomy in the group treated with MMC. Fibroblasts appear to be key cells mediating these effects.

## 1. Introduction

Tracheostomy, most commonly carried out in intensive care settings, is a life-saving tool for modern physicians [1,2]. In spite of its broad range of indications, there are only two absolute contraindications: significant skin or soft tissue infections and conditions leading to distorted anatomy. Tracheostomy is indicated for ventilator-dependent patients to facilitate ventilator weaning. Additionally, it allows convenient access for pulmonary toileting, reduces the need for sedatives, promotes patient comfort, reduces the risk of long-term sequelae of endotracheal tube placement, and potentiates intensive care unit (ICU) discharge [3].

Unfortunately, tracheostomy is also associated with appreciable morbidity, with reported complication rates of 8%–45% [3,4]. Tracheostomy-related complications include early and late complications. One of the most devastating late complications is benign tracheal stenosis, which can occur following prolonged intubation and tracheostomy.

Mitomycin-C (MMC) is an antitumor and antimitotic drug that is derived from *Streptomyces* *caespitosus* . It has an antineoplastic and angiogenesis properties that break DNA cross-linking like alkylating agents. At higher concentrations, it inhibits protein and RNA synthesis [5]. MMC inhibits in vitro fibroblast proliferation, causes apoptosis in fibroblasts, and prevents the formation of scars and fibrosis in humans. Although MMC can be toxic when administered systemically, the topical application prevents systemic toxicity [6]. Its effects have been tested during surgical treatments for pterygium, upper urinary tract urothelial tumors, endoscopic sinus procedures, maxillary antrostomy, and dacryocystorhinostomy [7]. We aimed to investigate the effects associated with topical application of MMC after conventional tracheostomy in a rabbit model.

## 2. Materials and methods

Twenty-four male, 24-week-old New Zealand White rabbits were used for this study and were randomly divided among 3 groups. All animals received humane care and were used in compliance with standards established by the European Convention for Animal Care and Use of Laboratory Animals. The rabbits were fed a standard pelleted diet and were allowed to access tap water ad libitum. The animals were housed in standard individual cages on a 12-h light/dark cycle at room temperature in a humidity-controlled environment. The local Animal Ethical Committee approved all study-related procedures. This study was approved and funded by the School of Medicine Animal Care and Investigational Committee at our institution.

###  2.1. Groups

Each of the three equal groups contained eight rabbits. Group 1 served as a control for tracheal measurements. After the tracheostomy, we applied either sterile saline (group 2) or MMC at 0.8 mg/mL (group 3) (Mitomycin-C Kyowa 10 mg/flk, Kyowa Hakko Kogyo Ltd. Tokyo, Japan) for 5 min.

### 2.2. Anesthesia

General anesthesia was induced with ketamine HCl (Ketanest, Pfizer Pharma GmbH, Karlsruhe, Germany), 15–20 mg/kg i.v. or 20–25 mg/kg i.m. moreover, maintained with xylazine (alfazyne 2%; Alfasan International. BV, Woerden, Netherlands) 0.5–1 mg/kg i.v. or 1–2 mg/kg i.m. If needed, standard doses of ketamine HCl or xylazine were repeated upon the emergence of reflex responses (pedal reflex, palpebral, and corneal reflexes) to maintain a constant anesthesia depth. Body temperature was monitored by inserting a heat probe into the ECG and rectum by the aid of needle electrodes. Heating lamps were used to keep the animals at 37 ± 5 °C body temperature during the surgical preparation and working periods. The mean anesthesia time was 12–15 min for each rabbit. No animals were treated with any local or systemic antibiotics.

### 2.3. Operative technique

**Figure 1:**
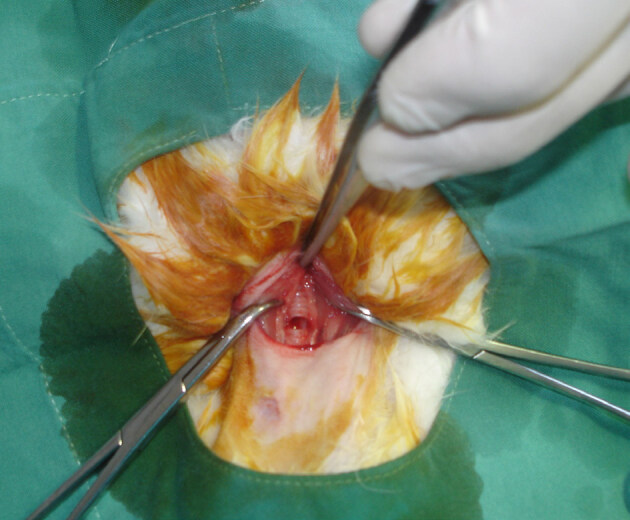
A horizontal incision was made under the cricoid at the level of trachea.

**Figure 2:**
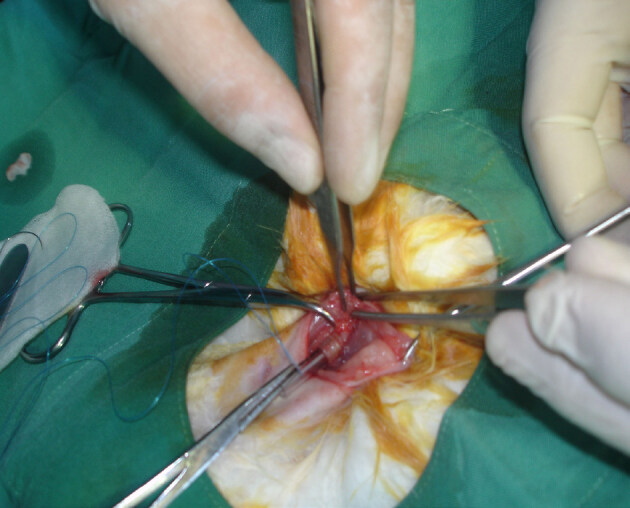
The tube obtained in sterile conditions from intubation set was introduced into the trachea and the tube fixed to the strap muscle.

Under general anesthesia, all rabbits were placed in the supine position on the operating table while breathing spontaneously. Their necks were shaved and cleaned with povidone-iodine solution. A vertical midline cervical incision performed over the larynx after infiltrating with 1% lidocaine and with 1:100000 epinephrine (Jetokain Simplex ampule; Adeka Pharmaceutical Company, İstanbul, Turkey). The strap muscles were divided along the median raphe to allow exposure of the larynx, cricoid, and superior trachea. A horizontal incision was made under the cricoid, at the level of 2nd or third 3rd rings (Figure 1). A sterile tube from the intubation set was introduced into the trachea and fixed to the strap muscle (Figure 2). Then, the skin was sutured with 4/0 silk suture. Classic tracheostomy was performed on 16 rabbits. Group 1 served as a control for tracheal measurements. After tracheostomy, group 2 rabbits had sterile saline applied topically, with cotton pledgets, around the tracheostomy tube site for 5 min. For group 3, MMC (0.8 mg/mL) was applied topically, using cotton pledgets, for 5 min.

### 2.4. Postoperative care and follow-up

Pain control in animals was provided with tradomol HCl (contramal, 100 mg, 2 mL, Abdi Ibrahim Ltd., Istanbul, Turkey) 1–2 mg/kg/day i.m. for 5 days during the postoperative period. The animals were followed-up for 3 weeks after surgery. All animals were painlessly euthanized with a lethal intravenous (IV) dose of nonbarbiturate anesthetic (ketamine/xylazine), according to instructions established by the latest report of the American Veterinary Medical Association (AVMA) Panel on Euthanasia. The anesthetic dose used was three times that necessary for euthanasia [8].

### 2.5. Pathological evaluation

**Figure 3:**
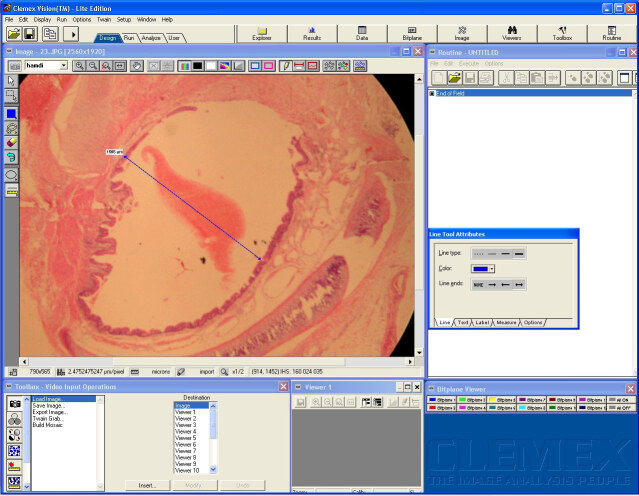
Tracheal lumen diameter is measured at the 20-fold magnified area in tissue samples by Clemex Vision Lite 3.5 image analysis system. The measurement is made on the narrowest line in the midline.

**Figure 4:**
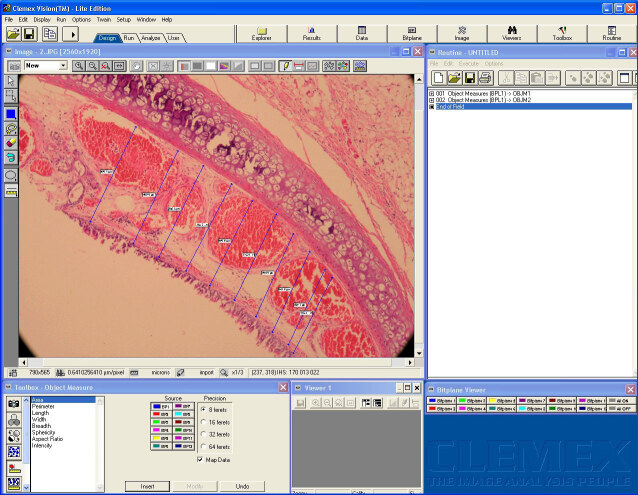
Subepithelial tissue thickness is measured at the 20-fold magnified area in tissue samples by the same image analysis system. The thickness is measured in a few places during the measurement, and the average is calculated automatically by the Clemex Vision Lite 3.5 image analysis system.

The larynx and trachea that were acquired from each subject were immediately fixed with 10% buffered formaldehyde. The tracheotomy sites of each specimen were cut transversely into 3 mm sections (5 sections per material). Samples were processed according to the autotechnician process, embedded in paraffin wax, and cut into 5 μm sections by a rotary microtome. Tissues were mounted on slides and stained with hematoxylen and eosin (H&E) and Masson’s trichrome. These tissues were examined under a Nikon Eclipse E400 light microscope (Nikon Corporation. Minato Ku, Tokyo Japan). The slides were photographed using Nikon Coolpix 5000 photographic attachment (Nikon Corporation). Photographs of the Nikon micrometer microscope slides (Nikon Stage Micrometer MBM11100) were also taken during the procedure. All photographs were then transferred to a computer and subjected to morphometric examination. The length was calibrated using a photograph of the Nikon micrometer microscope slide, which was taken under the same magnification. Tracheal lumen diameters (LD) (Figure 3) and subepithelial tissue thickness (SETT) (Figure 4) were measured with the Clemex Vision Lite 3.5 image analysis program (Clemex Technologies Inc. Longueuil, Quebec, Canada). We designated 0.25 mm square areas using the Clemex Vision Lite 3.5 image analysis system. Fibroblasts, inflammatory cells (IC), and capillary vessels (CV) were marked and automatically counted with the same image analysis system. The H&E sections were used to analyze IC. Other measurements were performed on Masson’s trichrome stained sections. Damaged cells were excluded from the evaluation. All slides were examined by the same pathologist, in a blinded fashion.

### 2.6. Statistical analysis

Data was assessed using the SPSS v.18.0 portable for Windows programme (SPSS Inc, Chicago, IL, USA). Data noted at 3 weeks posttracheostomy are expressed as medians and interquartile ranges. Between-group comparisons were made by the Kruskal–Wallis Test. The Mann–Whitney U-test was used to compare the control and saline/MMC groups. A p-value < 0.05 was used to indicate a significant difference.

## 3. Result

Regardless of the cause of death, dead animals were replaced with new ones. Twenty-four rabbits were included in our experiment, and all lived to the end of the 3 weeks. There were no instances of skin reaction, wound infections, or bleeding around the surgical sites during the perioperative period. No local complications attributable to MMC were encountered.

There was a statistically significant difference (p ˂ 0.035) between the groups in LD. In group 1, LD was 3198.8 (3027.3–3926.9). A maximum diameter loss of 2530.3 (1415.3–2897.5) was detected in group 2, and a loss of 3243.3 (2148.6–3771.9) was found in group 3. Because of the lack of between-group differences in LD between the MMC and control groups, it appears that MMC did not prevent lumen diameter loss.

Differences in SETT were statistically significant (p ˂ 0.006). In the control group, SETT was 168.3 (94.3–281.1). The highest increase in SETT was in group 2, with 516.1 (327.3–741.1). This was followed by group 3, with 298.9 (243.9366). Compared with the control group, SETT was increased by 3-fold in group 2 and 1.7-fold in group 3.

Between-group differences in IC were statistically significant (p ˂ 0.001). We detected 10 (6.5–16) ICs in the control group. The highest increase in IC was in group 2, with 229.5 (48.5–454), followed by group 3, with 34 (30.5–39). Compared with the control group, there was a 23-fold increase in IC for group 2, and a 3.4-fold increase in IC for group 3.

There were statistically significant between-group differences (p ˂ 0.001) in the CV. In the control group, we detected 4 (2–6) CVs. The highest increase in CV was group 2, with 19 (15– 29.5), followed by group 3, with 8 (6–9). Compared with the control group, CV was increased five-fold in group 2 and two-fold in group 3.

There were statistically significant between-group differences (p ˂ 0.001) in the number of fibroblasts. In the control group, we detected 5 (3.5–8) fibroblasts. The highest increase in fibroblasts was in group 2, with 30.5 (21–36), followed by group 3, with 10 (8.5–14) fibroblasts. Compared with the control group, fibroblasts were increased by six times for group 2, and 2.4 times for group 3.

There was a statistically significant difference between IC, CV, and fibroblasts in group 1 (control) and group 2 (tracheostomy + sterile saline). P-values were 0.007, 0, 042, and 0.037, respectively. LD (p = 0.730) and STT (p = 0.068) differences in the same groups could not be detected. LD, STT, IC, CV, and fibroblasts were significantly different in group 1 (control) and group 3 (tracheostomy MMC). P-values were 0.009, 0.024, 0.013, 0.014, and 0.023, respectively. In group 1 (tracheostomy sterile saline) and in group 3 (tracheostomy MMC), there was a statistically significant difference between IC, CV, and fibroblasts, but not LD and STT. P-values are 0.111, 0.111, 0.006, 0.006, and 0.02, respectively.

All the above values are listed in Table. MMC was more effective than saline for LD, SETT, IC, CV, and fibroblasts.

**Table:** Comparison of experimental group parameters.

	Group 1( control )	Group 2( tracheostomy + sterile saline)	Group 3( tracheostomy + MMC)	p- value
Tracheal lumen diameter*	3198.8 (3027.3–3926.9)	2530.3 (1415.3–2897.5)	3243.3 (2148.6–3771.9)	0.035
Subepithelial tissue thickness*	168.3 (94.3–281.1)	516.1 (327.3–741.1)	298.9 (243.9–366)	0.006
Inflammatory cells**/***	10 (6.5–16)	229.5 (48.5–454)	34 (30.5–39)	<0.001
Capillary vessels **/***	4 (2–6)	19 (15–29.5)	8 (6–9)	<0.001
Fibroblasts**/***	5 (3.5–8)	30.5 (21–36)	10 (8.5–14)	<0.001

*μm; **μm/pixel; *** 0.25 mm2/ cell.

## 4. Discussion

Surgery and trauma unavoidably cause scarring, particularly in circular organs. These processes including tracheostomy can incite complications, including the formation of fibrotic tissue such as stenosis [6]. Tracheal stenosis (TS) is a significant reduction in the tracheal lumen, life-threatening and largely preventable. Today, MMC is used successfully in various surgical fields, including ophthalmology, otorhinolaryngology, and urology to prevent scar formation. As shown in our study; fibroblasts are one of the critical cells in scar formation, but they are not unique. MMC can be used to prevent the development of antiproliferative activity and stenosis in a limited dose and time [6,9]. 

TS is a potentially life-threatening condition, it lowers the quality of life, and, for clinicians it is the most challenging complication encountered in airway surgery [10]. Thankfully, clinically significant stenosis is very rare and occurs in less than 2% of patients, however, its worldwide incidence remains unknown [11]. While several treatments have been used for tracheal stenosis, these tend to fail due to the new scar formation and restenosis through either the persistence of the chronic inflammatory processes that caused the initial stenosis or as an iatrogenic result of surgical intervention [12]. Because of the MMC’s effect on fibroblast proliferation in TS, many in vitro and in vivo studies are available. However, Wang et al. have not recommended its use because of increasing anastomosis complications after resection. In another study, Gangar et al. used MMC for 16 years, but it has not been proven to be useful. The result is that the utility of MMC remains hypothetical and its future role is unclear [13]. Wang et al. evaluated 263 patients with idiopathic subglottic stenosis, over a period of 42 years. MMC was administered to 27 patients (11.4%) in the form of endobronchial injection without the mention of dose and duration of administration, and steroids were given to 12 patients (5%) over 40 years. Importantly, steroids can lead to anastomotic complications, as noted in the literature [14]. Gangar et al. studied pediatric airway diseases, and in a randomized, double-blind, placebo-controlled study, they applied 0.4 mg/mL MMC for 2 min in 24 patients. Their study was terminated and the treatment considered invalid. In a similar study, 0.5 mg/mL topical MMC was administered to 26 patients, either one or two times, here, the double application was found to be more beneficial. However, the authors attribute this to their use in more serious and difficult situations [15].

MMC can be used topically in surgical wounds, areas where excessive fibrosis formation can lead to functional impairment. MMC solution-soaked cotton can be applied to the operative area, with optimum control of the location and duration of application. Although the ideal application time and the dose of MMC are still matters of debate, MMC has been successfully used as an adjunctive therapeutic agent to prevent excessive scarring in various surgical fields including ophthalmology, otorhinolaryngology, and urology. Most human studies used a topical dose of 2–10 mg/mL, which is due to its historic use in ophthalmological studies, and 0.1–0.4 mg/mL, in animal studies. The application time varied from 2 to 5 min. We decided to use MMC for the longest period (5 min) reported in the literature, and at twice the upper dose used in animal studies. We, however, agree that humans can safely use and heal across a wider field of less-sensitive tissues, over repeated doses, and in higher concentrations [15]. In vitro, MMC inhibited proliferation of human fibroblasts at 1.6 mg/mL. This effect of MMC increases with dose and, in animal models, in vivo cultures, and airway it is limited to 3 weeks. On skin, it is limited to 4 weeks [16]. Therefore, we limited our study period to 3 weeks.

Even though TS is a small research area, it can threaten and lower quality of life. There are countless animal experiments and patient series in the literature that used MMC. The studies affirming its usefulness are mostly retrospective and case presentation in nature. Low-doses and small patient cohorts have rarely been used prospectively. According to our best knowledge, only one English-language study examined SETT in response to topical application of MMC. Our experimental study on this subject will, therefore, be the second.

Wang et al. applied MMC to 27 patients (11.5%) over a 40-year research period. That is, less than one patient per year (0.675 patients per year); moreover, they did not specify the dose or form of administration. The efficacy of MMC is limited to 3 weeks, even in high doses, and has no effect in low-dose, heterogeneous, and small patient cohorts.

We used MMC to prevent the formation of scar tissue. In accordance with the literature there were statistically significant differences between the tracheostomy and control groups for LD (p = 0.035), CV (p = 0.06), SETT, fibroblasts, and the number of lymphocytes (p < 0.001). We feel these effects were due to the high dose used. In addition, topical application prevented MMC from inciting localized complications.

This study has some limitations. Firstly, as an experimental animal study, our research results could differ if human tissues are used, therefore, additional prospective studies are needed.

Moreover, wound healing modulation may prevent scar formation but additional studies are needed to prove this. Lastly, fibroblasts are key cells, but they are not the only cells involved in these processes. Realistically, a single dose of medication is likely insufficient for preventing scar formation. In the future, more powerful and long-term derivatives of MMC and/or new drugs should be studied since MMC is a medication that has a dose and time restriction for antiproliferative activity on fibroblasts.

## Disclaimers

This case was presented as a poster presentation in the American College of Chest Physicians (CHEST) Meetings October 24-28. Montréal, Québec, Canada; 2015; and published as a supplement CHEST Journal October 2015; 148 (4_meeting abstracts): 300A-300A. doi: 10.1378/chest.2281105. 

## References

[ref1] (2010). : complications in fresh postoperative and late postoperative settings. Clinical Pediatric Emergency Medicine.

[ref2] (2013). technique of tracheostomy. Current Problems in Surgery.

[ref3] (2014). Tracheostomy current surgical therapy: expert consult. Cameron JL.

[ref4] (2012). First do no harm: should routine tracheostomy after oral and maxillofacial oncological operations be abandoned?. British Journal of Oral and Maxillofacial Surgery.

[ref5] (2005). Increased expression of epidermal growth factor receptors in the tracheal epithelia after topical mitomycin-C in rabbits. Auris Nasus Larynx.

[ref6] (2010). Topical use of MMC in the upper aerodigestive tract: a review on the side effects. European Archives of Oto-Rhino-Laryngology.

[ref7] (2008). Effect of mitomycin in the surgical treatment of tracheal stenosis. Archives of Otolaryngology – Head & Neck Surgery.

[ref8] (2013). AVMA Guidelines for the Euthanasia of Animals: 2013 ed.

[ref9] (2017). Systematic review for surgical treatment of adult and adolescent laryngotracheal stenosis. Laryngoscope.

[ref10] (2009). Topical heparin: a promising agent for the prevention of tracheal stenosis in airway surgery. Journal of Surgical Research.

[ref11] (2014). Tracheal stenosis after intubation and/or tracheostomy. Egyptian Journal of Chest Diseases and Tuberculosis.

[ref12] (2005). The effects of mitomycin C and 5-fluorouracil/triamcinolone on fibrosis/scar tissue formation secondary to subglottic trauma (experimental study). American Journal of Otolaryngology.

[ref13] (2012). Management of complex benign post-tracheostomy tracheal stenosis with bronchoscopic insertion of silicon tracheal stents, in patients with failed or contraindicated surgical reconstruction of trachea. Journal of Thoracic Disease.

[ref14] (2013). Tracheal reconstruction for complex acute tracheal stenosis annals of medicine and surgery. Annals of Medicine and Surgery (London).

[ref15] (2000). Stents and sense. Annals of Thoracic Surgery.

[ref16] (2005). Management of benign stenosis of the large airways in the university. in 1998-2003. Respiration.

